# Laminectomy vs. laminoplasty for treating multi-segment cervical canal stenosis combined with central cord syndrome in the absence of fracture or dislocation: a retrospective study

**DOI:** 10.3389/fsurg.2026.1722910

**Published:** 2026-06-08

**Authors:** Qian Zhang, Rudan Guo, Jun Wang, Yuefen Wu, Shunyi Tong, Sanhua Fang, Xiaoling Yang

**Affiliations:** Department of Orthopedics, Lanxi People’s Hospital, Jinhua, Zhejiang, China

**Keywords:** canal stenosis, central cord syndrome without fracture or dislocation, efficacy comparison, laminectomy, laminoplasty, multi-segment, ossification of the posterior longitudinal ligament

## Abstract

**Background:**

Laminectomy fusion fixation (LF) and single open-door laminoplasty (LP) are common posterior surgeries for central cord syndrome without fracture/dislocation (CCSWOFD), yet a comprehensive comparison is lacking. This study compared outcomes of LF vs. LP for multi-segmental cervical canal stenosis with CCSWOFD.

**Methods:**

A retrospective analysis was conducted on 112 patients (LF group, *n* = 59; LP group, *n* = 53). Clinical and radiological outcomes were assessed. A *post-hoc* power analysis was performed, and multivariate logistic regression was used to identify factors associated with favorable outcomes, adjusting for key confounders.

**Results:**

The LF group demonstrated significantly better final Japanese Orthopaedic Association (JOA) scores, recovery rates (RR), intrinsic hand muscle strength (IHMS), and Brain and Spinal Injury Center (BASIC) scores compared to the LP group (*P* < 0.05). LF also achieved superior postoperative C2–7 Cobb angles despite reduced range of motion (ROM). The sagittal vertical axis (SVA) decreased significantly in the LF group but increased in the LP group. Notably, LF showed superior IHMS and RR outcomes in K-line (-) patients. Complication rates did not differ significantly between groups. Multivariate analysis identified higher preoperative JOA and IHMS as independent protective factors for favorable recovery, while LF was associated with non-significantly greater odds of good outcomes after adjusting for confounders.

**Conclusion:**

Both LF and LP effectively improved neurological function, alleviated pain, enhanced motor ability, and reduced spinal cord edema in CCSWOFD patients with multi-segment stenosis. In this retrospective cohort, laminectomy fusion fixation was associated with superior neurological and radiological outcomes compared to laminoplasty for multi-segment CCSWOFD, particularly for patients with coexisting ossification of the posterior longitudinal ligament and K-line (-).

## 1 Introduction

Central cord syndrome without fracture or dislocation (CCSWOFD) is commonly characterized by normal radiographs (1) or the absence of trauma on imaging (2). These injuries predominantly affect older adults with degenerative changes in their cervical spine and primarily occur due to excessive extension, compressing the narrowed spinal canal (3, 4). Approximately 8.7% of individuals aged 60 and above experience CCSWOFD in the United States (5), while it affects nearly 47% of all cases of cervical spinal cord damage in Japan (6). Moreover, there is a marked increase in the incidence rate of this type of partial injury in China's aging population.

Central cord syndrome is a commonly observed incomplete injury to the cervical spinal cord, frequently observed in the absence of fractures or dislocations. The clinical definition of central cord syndrome was initially proposed by Schneider et al. in 1954 (7). Despite the lack of visible fractures or dislocations in the cervical region, most patients present with pre-existing degenerative conditions like herniated discs, ossification of the posterior longitudinal ligament (OPLL), and hypertrophy of the ligamentum flavum (8). The clinical presentation entails varying degrees of paralysis in both upper and lower extremities, with a greater severity in the upper extremities. Additionally, patients may exhibit different levels of sphincter dysfunction and sensory impairment. When an external force is applied to the patients' neck region, cervical disc herniation, with or without osteophyte formation and OPLL, contributes to the narrowing of the spinal canal and the subsequent development of central cord syndrome.

Patients with central cord syndrome and neurological symptoms typically need surgical intervention rather than conservative therapy (9). There is an ongoing debate concerning the indications, timing, approaches, and prognostic factors for surgical treatment (10). Anterior or posterior cervical surgery may be conducted based on the extent of spinal cord injury and canal stenosis. Anterior procedures, such as anterior cervical discectomy and fusion (ACDF) and anterior cervical corpectomy and fusion (ACCF) are commonly employed; however, they are better options for patients with the involvement of less than three segments. As an advantage, these anterior procedures provide direct access to the affected area. They avoid major anterior neurovascular and visceral structures, such as the esophagus, trachea, carotid arteries, and recurrent laryngeal nerves. This approach allows for better visualization and easier removal of discs or bone fragments. Conversely, posterior approaches are more favorable for managing multi-segment cervical canal stenosis due to their lower risk of complications (9–11). This may be attributed to factors such as reduced intraoperative manipulation of vital structures, like blood vessels and esophagus. Minimizing these risks can improve patients' outcomes and expedite recovery (11).

Among posterior surgical options for patients with CCSWOFD, laminectomy fusion fixation and single open-door laminoplasty are widely employed. However, no comprehensive study compared these two surgical procedures. Therefore, this retrospective study was conducted to investigate the clinical outcomes of laminectomy fusion fixation and single open-door laminoplasty for treating multi-segment spinal cord stenosis combined with CCSWOFD.

## 2 Methods

### 2.1 General information

From January 2012 to December 2021, 112 patients with central cord syndrome but without fracture or dislocation were selected for this study. The patients underwent either posterior laminectomy fusion or single open-door laminoplasty at Department of Spine Surgery, Lanxi People's Hospital. Preoperative anteroposterior and lateral x-ray films, CT scans, and magnetic resonance imaging (MRI) of the cervical spine were obtained to measure the extent of spinal cord compression and injury. The inclusion criteria were as follows: (1) presence of multi-segment cervical canal stenosis involving three or more segments, evidenced by a spinal canal anteroposterior diameter of less than 12 mm on imaging; (2) absence of severe cervical kyphosis (Cobb angle > 40°); (3) presence of symptoms related to central cord injury; and (4) availability of complete preoperative and postoperative data with a minimum follow-up duration of at least 24 months. The exclusion criteria were as follows: (1) patients with spinal canal stenosis involving only one or two segments and those presenting with evident cervical kyphosis (˃40°) unsuitable for posterior surgeries; (2) patients with a history of surgery on the corresponding segment, combined spinal deformity, tumor or infection; (3) individuals with concurrent diseases affecting neurological function assessment, such as motor neuron disease, Parkinson's disease, cerebral palsy, and cerebral infarction; and (4) patients with incomplete postoperative follow-up data or a follow-up time less than two years. This study was conducted in accordance with the Declaration of Helsinki and approved by the Ethics Committee of Lanxi People's Hospital (20240929001).

### 2.2 Surgical methods

The patients underwent general anesthesia and were kept in a prone position, with head facing downward on the head frame and abdomen elevated. No pressure was applied to the eyes, and standard sterilization procedures were conducted to prepare the surgical area. A posterior median incision was performed. All patients underwent surgery within 48–72 h of admission.

A 12 cm incision was made from the lower edge of the C2 spinous process to the upper edge of the C7 spinous process in the laminoplasty (LP) group. Sequentially, an incision was made on the skin, subcutaneous tissue, and fascial layers. The paravertebral muscles on both sides were dissected along the spinous process. Bilateral laminae of C3–C6 up to the facet joint were medially exposed. After confirming decompression segments through C-arm x-ray fluoroscopy, bone grafting was conducted by removing the spinous processes of C3–C6. A groove was created at the inner edge of each facet joint on both sides (C3–C6) for careful grinding with a drill that selectively removed only the outer cortical bone while preserving the integrity of the inner cortical bone. A laminectomy rongeur was used to open and expose laminae from C3–C6 on one side. Subsequently, steel plates measuring 8–12 mm in length were individually placed at C3–C6 levels. They were secured with screws for internal fixation. Intraoperative fluoroscopy confirmed the accurate positioning of internal fixation devices. The surgical site was irrigated with high amounts of normal saline solution for effective cleansing and controlling bleeding. Once active bleeding stopped under observation, strips cut from spinous process bones were implanted alongside portal shafts.

A 15 cm longitudinal incision was made along the spinous process in the laminectomy and fusion (LF) group. The skin, subcutaneous tissue, and posterior cervical fascia were incised, and the paravertebral muscle was dissected layer by layer. Fluoroscopy was used to confirm the accurate positioning of screws. The connecting rod of appropriate length was pre-bent and positioned at the tail of each screw to match the physiological curvature. The physiological lordosis of the cervical spine was restored by extending the neck backward. The edges of the bilateral lamina were gradually polished using grinding drills until reaching the inner cortical bone. Residual connective bone tissue and ligamentum flavum on the polished side of laminae were meticulously removed using forceps in an “uncapping” manner. Subsequently, the C3–C6 laminae were fully exposed while completely eliminating any pressure factors during surgery. Exploration revealed an intact dura mater with noticeable bulging and normal pulsation. Thorough polishing was conducted on bilateral lateral masses of C3–C6. The removed lamina was prepared into 2–3 mm granular bone grafts for fusion. All patients underwent surgery at the C3–6 segment to maximize posterior displacement of the spinal cord while minimizing the risk of axial symptoms due to increased exposure of C7.

Both LF and LP can be used in the treatment of multisegmental cervical stenosis. To minimize selection bias and clarify clinical decision-making, a standardized surgical protocol was followed at our institution. LF was generally preferred for patients with (1) significant cervical kyphosis (Cobb angle > 10°), (2) evidence of interbody instability on dynamic radiographs, or (3) an OPLL occupying ratio exceeding 60%. Conversely, LP was typically chosen for patients with preserved cervical lordosis (Cobb angle ≤ 10°), no evidence of instability, and an OPLL occupying ratio ≤ 60% (12, 13). These criteria were applied consistently throughout the study period.

### 2.3 Postoperative management

Postoperative vital signs were closely monitored, and methylprednisolone and antibiotics were administered for 3 days. This treatment regimen was administered to all patients throughout the retrospective study period. The timing of drainage tube removal was determined based on the color and volume of wound discharge. To promote optimal healing, cervical braces were used for 4 weeks, during which gradual introduction of gentle movements, such as nodding and turning the head, followed by the removal of drainage tubes.

### 2.4 Surgical complications

Patients were monitored for a minimum duration of 24 months (LF group 32.5 ± 7.2, LP group 33.5 ± 8.7) to monitor the incidence of surgical site infection, cerebrospinal fluid leakage, symptoms associated with the axial region, fifth cervical nerve root paralysis, spinal epidural hematoma, and other potential complications.

### 2.5 Assessment of neurological function

The neurological function of patients was assessed using the Japanese Orthopaedic Association (JOA) score preoperatively and in the final follow-up period. The rate of neurological recovery (RR) was determined using the following formula: (final JOA score—preoperative JOA score)/[17 (maximum score)—preoperative JOA score] (14). Following Yamazaki et al.'s method, a favorable prognosis was defined as a JOA score improvement rate >50%, while an unfavorable prognosis was defined as a JOA score improvement rate ≤ 50% (15).

### 2.6 Assessment of pain

The neck and upper limb pain was assessed before the surgery and during the final follow-up using the Visual Analogue Scale (VAS) score. Pain intensity was determined based on a numerical scale ranging from 0 to 10, with higher scores indicating more severe pain (16).

### 2.7 Assessment of motor function

The ASI motor score, a component of the American Spinal Injury Association Impairment Scale, is a standardized assessment method used to evaluate motor strength by examining myotomes (17). Given its predominant role in upper limb functionality, especially the hands and grip strength (18), we employed scores for intrinsic hand muscle strength (IHMS) to assess hand mobility, including bilateral digital flexor muscle strength and little finger abductor muscle strength. The evaluation utilizes a universally recognized six-point scale (ranging from 0 to 5) to assess the bilateral motor strength of each muscle group, with higher scores indicating greater hand strength (19, 20). The muscle strength of the deep flexor muscles, superficial flexor muscles of the fingers, and abductor muscle of the little finger in both hands is traditionally graded on a 0 - to - 5 scale, with each grade corresponding to one point. The total possible score across all muscle groups is 110 points. A score of 0 represents the absence of muscle contraction, while 5 indicates normal strength. A postoperative muscle strength grade of 3 or higher is considered indicative of satisfactory functional recovery, and a total composite score of 66 or above is regarded as favorable.

### 2.8 Assessment of intraparenchymal cord injury

The Brain and Spinal Injury Center (BASIC) score was used to assess the extent of intramedullary high-signal and intraparenchymal cord injury. Briefly, a spinal cord injury (SCI) with a BASIC score of 0 indicated normal T2 relaxivity of the spinal cord without any discernible pathological intramedullary signal. A BASIC score of 1 represented a case with confined pathological T2 hyperintensity primarily in the gray matter of the spinal cord. If the pathological intramedullary T2 hyperintensity extended beyond the margins of central gray matter and obscured the boundaries between gray and white matter, it received a BASIC score of 2. In contrast, if the entire transverse extent of the spinal cord was affected by pathological T2 hyperintensity without any remaining normal-appearing white matter, it received a BASIC score of 3. Finally, an SCI with a BASIC score of 4 referred to an injury classified as a BASIC Score of 3 but also exhibited additional distinct areas of macroscopic intramedullary hemorrhage resulting in hypointense signals on T2 imaging (21).

### 2.9 Assessment of employability

The assessment of daily functional recovery and employability level was conducted using the Indian modifications of the (imNurick) scale, which is a standardized six-point grading system ranging from 0 to 5. Grade 0 represents signs or symptoms of root involvement, without any evidence of spinal cord disease. Grade 1 represents the presence of spinal cord disease with no difficulties in movement. Grade 2 represents mild challenges in ambulation, yet the individual can independently rise from a squatting position or sitting on the ground without external support. Grade 3 reflects moderate difficulty in walking and necessitates external support when transitioning from squatting or sitting positions. Grade 4 denotes dependence on assistance from another person or assistive device for ambulation. Grade 5 implies being confined to a wheelchair or being bedridden (22). A higher grade implies poorer employability and worse daily functioning.

### 2.10 Imaging assessment

**Figure 1:**
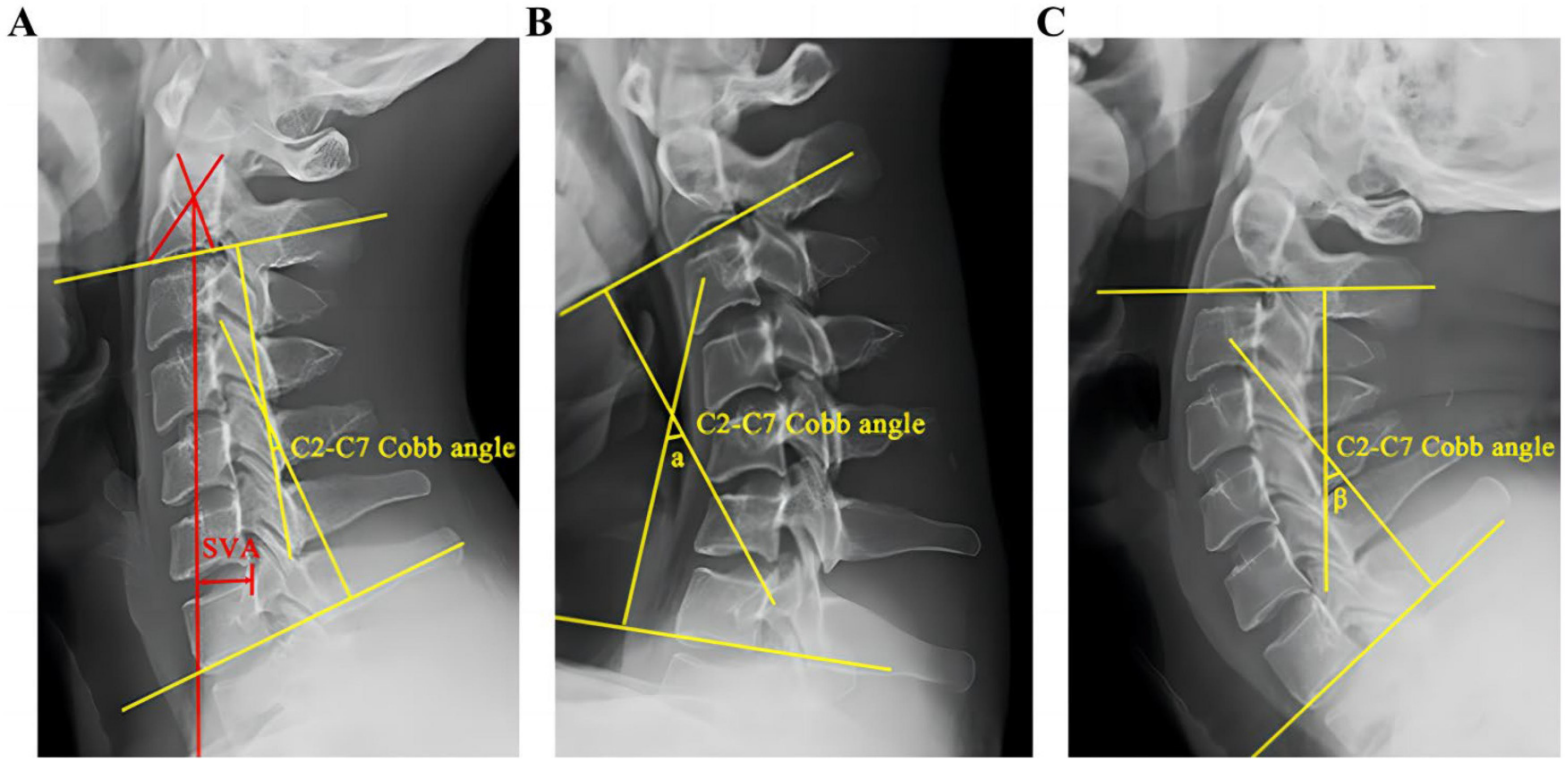
**(A)** The schematic representation of C2–7 SVA and C2–C7 Cobb Angle. C2–7 SVA refers to the distance between the posterosuperior corner of C7 and a plumb line dropped from the center of C2 vertebral body, while C2–C7 Cobb angle is measured between the lower endplates of C2 and C7 on a standard cervical lateral x-ray. Moving on to Pictures **(B,C)**, these diagrams depict the ROM in the cervical spine. The flexion angle, denoted as α, assumes a negative value in the presence of a reverse curvature. Conversely, the hyperextension angle, labeled as α, assumes a negative value.

**Figure 2:**
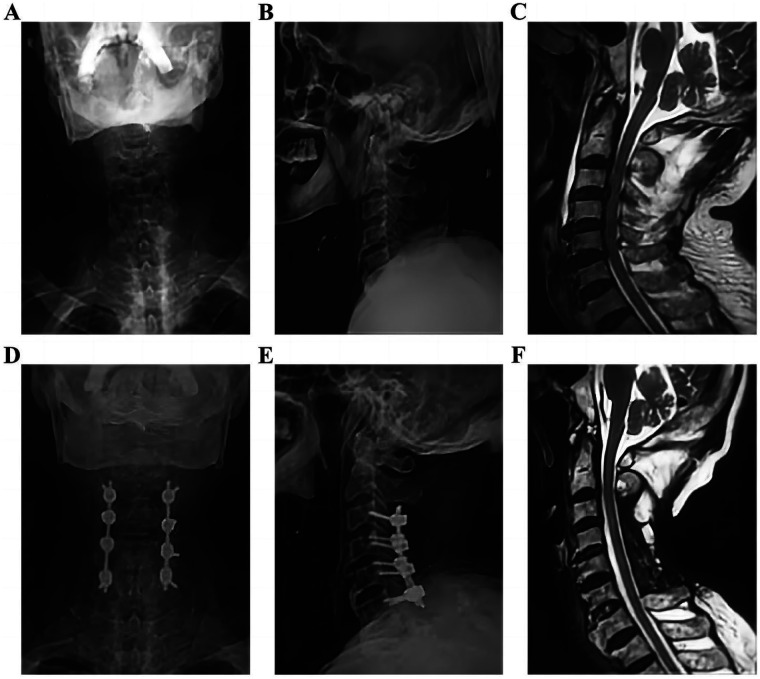
Patient 1, a 61-year-old male, presented with cervical pain and experienced bilateral upper limb numbness and weakness following an injury that occurred 3 h before admission. The diagnosis was multilevel cervical stenosis accompanied by central canal syndrome. To address this condition, the patient underwent posterior laminectomy fusion fixation. **(A,B)** demonstrate the presence of normal cervical lordosis without any significant narrowing in the intervertebral spaces. **(C)** displays multilevel cervical stenosis along with high signal intensity detected in the C3/4 segment of the cord on preoperative cervical MRI scans. **(D,E)** exhibit postoperative lateral and anterior-posterior x-rays of the cervical spine, indicating satisfactory positioning of internal fixation devices. **(F)** represents a postoperative cervical MRI scan revealing noticeable posterior displacement of the cord without substantial compression.

**Figure 3:**
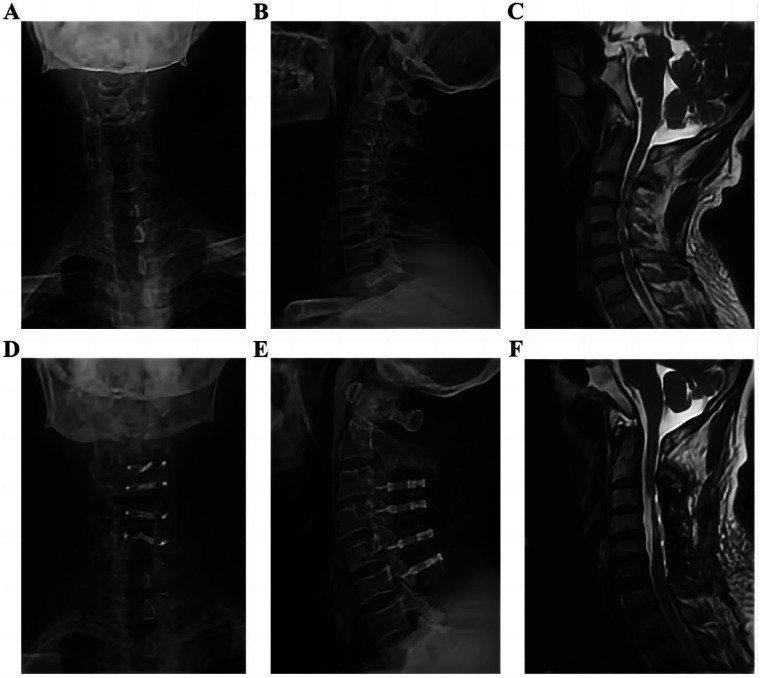
Patient 2, a 57-year-old male, came to the hospital with acute pain and weakness in both upper extremities after a trauma that occurred three hours before admission. He had a history of cervical spinal stenosis resulting in central canal syndrome at multiple levels. A posterior single-door laminoplasty was conducted, and radiographs [**(A,B)** films] demonstrated normal cervical lordosis without significant intervertebral space narrowing. Preoperative cervical MRI **(C)** revealed multiple instances of cervical spinal stenosis accompanied by high signal changes in the C3–5 spinal cord region. Postoperatively, radiographs [**(D,E)** films] showed satisfactory positioning of internal fixation devices, and postoperative cervical MRI [**(F)** film] revealed noticeable posterior displacement of the spinal cord without significant compression.

Cervical sagittal balance index (C2–7 SVA): The C2–7 SVA index provides invaluable insights into the sagittal balance and degree of anteversion in the cervical spine. To quantify this index, we precisely measured the vertical distance between the posterior-superior edge of C7 vertebra and a vertically aligned line passing through the center of the second cervical vertebra on a lateral x-ray image (Figure 1A). A higher C2–7 SVA value indicated a potential compromise in the sagittal balance of the cervical spine (23). Range of motion (ROM): Cervical ROM was determined based on dynamic x-ray imaging during neck flexion and hyperextension, with a specific focus on measuring the C2–7 Cobb Angle. The angle β represents flexion, where a negative value indicates reverse motion in the cervical spine (CS). An angle reflecting loss was obtained by subtracting postoperative ROM from preoperative ROM (Figures 1B,C). C2g.B.Cgle reflex: This measurement determined an angle between the lower endplates of C2 and C7 on a standard lateral x-ray film (Figure 1A). Two representative cases are shown in Figures 2A–F and Figures 3A–F.

### 2.11 Statistical analysis

SPSS software (SPSS Inc., Chicago, IL, USA) version 22.0 was used for statistical analysis. Measurement data are presented as mean and standard deviation. They were analyzed using t-tests. Count data are expressed as percentages and analyzed using *χ*² tests. A multivariate logistic regression analysis was used to identify factors associated with favorable postoperative outcomes. Favorable JOA recovery was defined as a final JOA score ≥ 10 points, a threshold chosen based on previous studies indicating it represents a clinically meaningful improvement (24); this definition was used consistently in the regression analysis. Favorable IHMS recovery was defined as a final IHMS score ≥ 66 points, consistent with the definition of satisfactory functional recovery in Section 2.7. The selection of independent variables (preoperative JOA, age, preoperative IHMS, preoperative ROM, surgical method, and preoperative C2–7 Cobb angle) was based on clinical relevance and findings from prior literature. Table 1 summarizes the covariates included in the multivariable logistic regression models, along with their definitions, measurements, and the rationale for inclusion. No adjustment for multiple comparisons was made, as the analyses were considered exploratory. Statistical significance was considered when *P*-value < 0.05. A post - hoc power analysis (presented in the supplemental material) was carried out using G*Power software (version 3.1). Based on the observed difference in the final JOA scores between the groups (with a mean difference of 2.30 and a pooled standard deviation of 1.19), the achieved sample size (*n* = 112) offered 92% power to detect a significant difference at *α* = 0.05, indicating that the study was adequately powered for its primary outcome.

**Table 1:** Covariates included in the multivariable logistic regression models.

Covariate	Variable type	Definition/Measurement	Rationale for inclusion
Surgical method	Categorical	LF = 1, LP = 0	Primary exposure variable of interest
Preoperative JOA score	Continuous	Japanese Orthopaedic Association score (0–17), higher score indicates better neurological function	Strongest known predictor of postoperative neurological recovery
Age	Continuous	Patient age at surgery (years)	Known independent predictor of neural plasticity and recovery
Preoperative IHMS score	Continuous	Intrinsic hand muscle strength score (0–110), higher score indicates better hand motor function	Directly correlates with the primary outcome (IHMS recovery)
Preoperative ROM (C2–7)	Continuous	Cervical range of motion from C2 to C7 (°), measured on dynamic radiographs	Reflects baseline cervical mobility, may affect functional outcomes
Preoperative C2–7 Cobb angle	Continuous	Cervical lordosis angle from C2 to C7 (°), measured on standing lateral radiographs	Key determinant of posterior surgical approach selection; affects sagittal balance

JOA, Japanese orthopaedic association; IHMS, intrinsic hand muscle strength; ROM, range of motion; LF, laminectomy fusion fixation; LP, laminoplasty.

## 3 Results

The LF group comprised 59 cases, including 31 males and 28 females, whose ages ranged from 39 to 75 (with an average age of 58.75 ± 8.72 years). In this group, there were 10 patients with multi-segment cervical disc herniation, 23 patients with OPLL and K-line (−), and 26 patients with OPLL and K-line (+). Of them, 5 had diabetes and 8 had hypertension. The LP group consisted of 53 cases, including 27 males and 26 females whose ages ranged from 28 to 77 years (with a mean age of 60.72 ± 9.45 years). Among them, 12 patients had multi-segment cervical disc herniation, 18 had OPLL with K-line (−), and 23 had OPLL with K-line (+). Moreover, 4 individuals had diabetes while 6 suffered from hypertension (Table 2). No significant differences were observed between the two groups in terms of gender, age, smoking status, diabetes, hypertension, number of disc herniations, number of ossified vertebrae, length of high-intensity signal in spinal cord, preoperative cervical lordosis, OPLL occupying ratio or intramedullary high-signal lesions (*P* > 0.05) (Table 2).

**Table 2:** Comparison of preoperative data of patients between the two groups.

Parameters	LF group	LP group	*P* value
Age (years)	58.75 ± 8.72	60.72 ± 9.45	0.365
Gender (male/female)	31/28	27/26	0.826
Smoking status (yes/no)	11/48	9/44	0.728
Diabetes mellitus (*n*, %)	5 (8.5%)	4 (7.6%)	0.175
Hypertension (*n*, %)	8 (13.6%)	6 (11.4%)	0.261
Number of disc herniation (*n*)	3.79 ± 0.58	3.68 ± 0.46	0.185
Intramedullary high-signal lesion (*n*)	1.98 ± 0.76	1.87 ± 0.65	0.126
Length of high-intensity signal in spinal cord (mm)	24.47 ± 8.26	22.10 ± 09.80	0.368
Preoperative cervical lordosis number (*n*%)	(23, 38.9%)	(18, 33.7%)	0.295
OPLL occupying ratio			
0%–30% (*n*)	29	24	
30%–60% (*n*)	20	17	
Number of ossified vertebra	3.93 ± 0.42	3.87 ± 0.56	0.205
OPLL type according to K-line (*n*, %)			
K-line (+)	(26, 44.1%)	(23, 43.4%)	
K-line (−)	(23, 38.9%)	(18, 33.7%)	

The duration of surgical procedures, overall blood loss, length of hospital stays, and number of opened lamina in each group are presented in Table 3. The two groups did not exhibit any significant differences in terms of overall blood loss (*P* = 0.526), length of hospitalization (*P* = 0.428), and number of opened laminae (*P* = 0.285). However, the LP group exhibited a shorter duration of surgery than the LF group (*P* = 0.03).

**Table 3:** Comparison of perioperative parameters between the two groups.

Parameters	LF group	LP group	*P* value
Operation duration (min)	188.3 ± 36.28	153.79 ± 22.37	0.03
Total blood loss (mL)	498.76 ± 315.69	423.56 ± 265.15	0.526
Length of hospital stay (day)	14.56 ± 3.76	13.85 ± 4.05	0.428
Number of opened lamina (*n*)	4.82 ± 0.98	4.38 ± 0.45	0.285

Table 4 presents the clinical outcomes and recovery of intraparenchymal cord injuries. No significant differences were observed between the two groups in terms of preoperative JOA score, VAS score, IHMS score, BASIC score, and RR (*P* > 0.05). However, after surgery, both groups exhibited significant improvements in all parameters (*P* < 0.05). The LF group demonstrated significantly superior JOA scores, IHMS scores, BASIC scores, and RR compared to the LP group (*P* < 0.05).

**Table 4:** Comparison of clinical outcomes at last follow-up.

Parameters	Point-in-time	LP group	LF group	*P* value	95% CI
JOA score	Before surgery	6.35 ± 1.12	6.98 ± 0.89	0.653	
	Last follow-up	10.68 ± 1.13	12.98 ± 1.25	0.015	0.325–0.985
	*P* value	*P* < 0.001	*P* < 0. 001		
VAS score	Before surgery	6.75 ± 1.23	7.03 ± 1.45	0.682	
	Last follow-up	2.33 ± 0.63	2.07 ± 0.52	0.857	0.257–2.355
	*P* value	*P* < 0. 001	*P* < 0.001		
IHMS score	Before surgery	49.75 ± 21.23	51.03 ± 20.45	0.849	
	Last follow-up	79.33 ± 19.63	91.07 ± 18.52	0.018	1.352–1.885
	*P* value	*P* < 0. 001	*P* < 0.001		
BASIC score	Before surgery	2.1 ± 0.6	2.2 ± 0.5	0.568	
	Last follow-up	0.8 ± 0.3	0.4 ± 0.2	0.032	1.463–1.609
	*P* value	0.035	0.041		
imNurick scale score	Before surgery	2.69 ± 0.31	2.35 ± 0.32	0.668	
	Last follow-up	1.52 ± 0.23	1.27 ± 0.19	0.462	0.113–1.252
	*P* value	0.015	0.021		
RR (%)	Last follow-up	52.22 ± 11.67	68.23 ± 8.23	*P* < 0. 001	1.625–1.789

JOA, Japanese orthopaedic association; VAS, visual analogue scale; IHMS, intrinsic hand muscle strength; BASIC, brain and spinal injury center; ImNurick, Indian modification of Nurick; RR, recovery rate.

The C2–7 SVA and ROM of the LF group and LP group were compared preoperatively and at the last follow-up (Table 5). The preoperative C2–7 SVA was comparable between the two groups (*P* > 0.05). However, at the last follow-up, C2–7 SVA significantly increased in the LP group and decreased in the LF group, indicating a significant difference between the two groups (*P* < 0.05). Regarding preoperative ROM, there was no significant difference between the LF and LP groups before surgery (*P* > 0.05). However, at the final follow-up, ROM decreased in both groups. Furthermore, there was a significant difference between the outcomes of the two groups (*P* < 0.05). Before surgery, there were no notable differences between the LF and LP groups in terms of preoperative C2–7 Cobb angle (*P* > 0.05). At the final follow-up, this angle increased to some extent in the LF group while decreasing slightly in the LP group, highlighting a significant difference between the two groups (*P* < 0.05).

**Table 5:** Comparison of radiological outcomes between the two groups.

Parameters	Point-in-time	LF group	LP group	*P* value	95% CI
C2–7 Cobb angle (°)	Before surgery	11.73 ± 1.72	12.65 ± 1.57	0.489	0.203–0.375
	Last follow-up	17.32 ± 1.78	10.18 ± 1.45	*P* < 0. 001	
	*P* value	*P* < 0. 001	0.018		
C2–7 SVA	Before surgery	20.93 ± 4.20	21.32 ± 3.18	0.567	1.257–1.596
	Last follow-up	18.05 ± 3.82	23.41 ± 2.78	0.008	
	*P* value	0.032	0.037		
ROM (°)	Before surgery	26.58 ± 3.50	27.06 ± 3.72	0.725	0.385–0.826
	Last follow-up	14.32 ± 3.78	24.79 ± 2.86	*P* < 0. 001	
	*P* value	*P* < 0. 001	0.023		

SVA, sagittal vertical axis; ROM, range of motion.

The surgical indications for LF and LP were investigated in our study, where patients with OPLL were classified into subgroups based on their K-line status (Table 6). Our results indicated that in the presence of a positive K-line, there were no significant differences between the two groups in terms of the final JOA score and IHMS score (*P* = 0.125 and *P* = 0.327, respectively). However, the LF group exhibited significantly superior outcomes in terms of RR compared to the LP group (*P* = 0.029). Conversely, in the presence of a negative K-line, we discovered significantly higher final JOA scores, IHMS scores, and RR in the LF group compared to the LP group (*P* = 0.016, *P* = 0.019, and *P* = 0.018, respectively).

**Table 6:** Comparison of clinical outcomes between the two groups based on K-line.

Features	Preoperative JOA (score)	Final JOA (score)	Preoperative VAS (score)	Final VAS (score)	Preoperative IHMS (score)	Final IHMS (score)	RR (%)
K-line (+) (inter. *P* value)	0.516	0.125	0.425	0.137	0.482	0.327	0.031
LFG = 26	7.75 ± 1.16	11.82 ± 1.05	6.85 ± 1.37	2.91 ± 0.85	51.15 ± 21.12	107.25 ± 21.12	57.35 ± 1.97
LPG = 23	7.98 ± 1.22	12.05 ± 0.92	6.92 ± 1.41	3.07 ± 1.12	49.35 ± 17.12	102.13 ± 19.12	36.35 ± 0.12
K-line (−) (inter. *P* value)	0.852	0.016	0.726	0.132	0.728	0.019	0.018
LFG = 23	8.35 ± 1.03	12.39 ± 0.92	6.72 ± 1.07	3.09 ± 0.72	46.17 ± 1.18	100.28 ± 20.49	37.21 ± 1.12
LPG = 18	8.16 ± 0.79	9.76 ± 1.17	6.82 ± 1.12	3.15 ± 1.03	47.36 ± 1.07	77.42 ± 11.73	19.45 ± 1.12

LFG, laminectomy fusion fixation group; LPG, laminoplasty group.

The incidence of postoperative complications was compared between the two groups (Table 7), revealing similar rates of incision site infection, cerebrospinal fluid leakage, axial symptoms, C5 nerve root palsy, and symptomatic spinal epidural hematoma (SEH) (*P* > 0.05). Furthermore, there were no significant differences between the two groups in terms of overall complication rates (*P* > 0.05).

**Table 7:** Comparison of postoperative complications between the two groups.

Complications	LF group	LP group	*χ*^2^ value	*P* value
SSI	1	1	0.008	1.000
CSF leakage	3	2	4.119	0.248
Axial symptoms	4	3	5.829	0.418
C5 palsy	5	4	7.374	0.612
Symptomatic SEH	1	1	0.008	1.000
Total	16	14	0.347	0.083

CSF, cerebrospinal fluid; SSI, surgical site infection; SEH, spinal epidural hematoma.

The findings from a multivariate logistic regression analysis of factors influencing good postoperative JOA (≥10 points) and IHMS (≥66 points) recovery are presented in Tables 8, 9. Multivariate logistic regression analysis identified higher preoperative JOA score (OR = 1.422, 95% CI: 1.158–1.746, *P* < 0.001) and higher preoperative IHMS score (OR = 1.469, 95% CI: 1.166–1.852, *P* = 0.001) as independent protective factors for favorable JOA recovery (Table 8). For favorable IHMS recovery, higher preoperative JOA (OR = 1.530, 95% CI: 1.167–2.006, *P* = 0.002) and higher preoperative IHMS (OR = 1.696, 95% CI: 1.258–2.286, *P* < 0.001) were also independent protective factors (Table 9). After adjusting for potential confounding factors, patients who underwent LF surgery had a 1.353-fold and 1.330-fold greater likelihood of achieving favorable postoperative JOA and IHMS recovery, respectively, compared to those who underwent LP surgery; however, these associations did not reach statistical significance (*P* = 0.146 for JOA, *P* = 0.225 for IHMS).

**Table 8:** The results of multivariate logistic regression analysis of factors affecting good postoperative JOA recovery (≥10 points).

Independent variable	β	Standard error	Wald value	OR	95% CI	*P* value
Preoperative JOA	0.352	0.105	11.23	1.422	1.158–1.746	<0.001
Age	−0.035	0.025	1.96	0.966	0.920–1.014	0.162
Preoperative IHMS	0.385	0.118	10.64	1.469	1.166–1.852	0.001
Preoperative ROM	0.052	0.048	1.17	1.053	0.959–1.157	0.279
Surgical method (LF vs. LP)	0.302	0.208	2.11	1.353	0.900–2.034	0.146
Preoperative C2–7 Cobb angle	0.125	0.098	1.63	1.133	0.935–1.373	0.202

JOA, Japanese orthopaedic association; IHMS, intrinsic hand muscle strength; ROM, range of motion.

**Table 9:** The results of multivariate logistic regression analysis of factors affecting good postoperative IHMS recovery (≥66 points).

Independent variable	β	Standard error	Wald value	OR	95% CI	*P* value
Preoperative JOA	0.425	0.138	9.48	1.530	1.167–2.006	0.002
Age	−0.042	0.028	2.25	0.959	0.908–1.013	0.134
Preoperative IHMS	0.528	0.152	12.06	1.696	1.258–2.286	<0.001
Preoperative ROM	0.038	0.052	0.53	1.039	0.938–1.150	0.465
Surgical method (LF vs. LP)	0.285	0.235	1.47	1.330	0.839–2.108	0.225
Preoperative C2–7 Cobb angle	0.098	0.112	0.77	1.103	0.886–1.373	0.381

JOA, Japanese orthopaedic association; IHMS: intrinsic hand muscle strength; ROM, range of motion.

## 4 Discussion

Our findings suggested that both laminectomy and laminoplasty can effectively improve neurological symptoms, reduce pain, enhance motor ability and employability, and relieve edema or hemorrhage of the cervical cord. Compared to preoperative levels, the final JOA scores and VAS scores of both groups significantly improved, suggesting a marked improvement in neurological function and pain relief. Furthermore, the LF group exhibited greater JOA scores and a higher rate of neurological recovery during follow-up (*P* < 0.05). This phenomenon can be explained by multi-level cervical canal stenosis, which can lead to spinal cord compression due to multiple protruding cervical intervertebral discs or OPLL at various locations and different degrees of relative displacement or angle between vertebral bodies during hyperextension or flexion of the cervical spine. Therefore, there will be an increased instantaneous pressure on the spinal cord, leading to more severe injury. Laminectomy decompression and fusion with a lateral mass fixation system can help maintain the normal lordotic physiological curvature of the cervical spine while controlling the intervertebral movement of the ossified segments. This strategy prevents late kyphosis and allows for better posterior shift and adequate decompression of the cervical cord. These findings suggest that posterior laminectomy with fusion may contribute to better long-term neurological function compared to laminoplasty in this cohort.

The assessment of the natural curvature of the cervical spine usually includes determining values, such as the C2–7 sagittal vertical axis (C2–7SVA), and measuring the Cobb angle between C2 and C7 (25). Dynamic x-ray films are used to assess cervical range of motion (ROM) by quantifying flexion and hyperextension angles at the level of C2–C7. The cumulative value obtained from these measurements reflects the extent of movement in this region. Kim et al. (26) have demonstrated that increased cervical lordosis is associated with decreased C2–7SVA values, while decreased lordotic curvature or increased kyphosis is associated with elevated C2–7SVA values. In our study, patients who underwent laminoplasty demonstrated a significant increase in their C2–7SVA values; however, there was a simultaneous decrease in both ROM and the Cobb angle encompassing C3–C7. In contrast, patients who underwent LF exhibited a notable increase in the C2–7 Cobb angle but a decrease in ROM and C2–7SVA at the final follow-up. The maintenance of lordotic physiological curvature through rigid fixation in the LF group, and unintended autofusion along the lateral margin of lamina can be attributed to this phenomenon. However, inadequate rigid fixation and paravertebral muscle dissection during LP can gradually lead to the development of sagittal imbalance in the cervical spine, thereby decreasing the C2–C7 Cobb angle. Additionally, lateral mass screw fixation in the LF group better restores the patient's physiological cervical lordosis, thereby achieving a larger Cobb angle and smaller SVA, maintaining superior sagittal balance (12).

The scores of IHMS significantly improved (*P* < 0.05) in both groups, indicating a substantial increase in patients' capacity for physical activity after either laminectomy or laminoplasty. However, the LF group demonstrated superior performance compared to the LP group by achieving significantly higher final scores. Xu L et al. (27) suggested that the ASI motor score upon admission can serve as a prognostic indicator for patients with CCS. However, this composite score includes both upper and lower extremities. Nevertheless, CCS primarily manifests with severe symptoms that mainly affect the upper limbs rather than the lower limbs, thus highlighting its predominant effect on the dysfunction of the upper extremities. Therefore, relying solely on the ASI motor score may not accurately predict patients' prognosis. The underlying pathogenesis of CCS lies in the anatomical distribution of corticospinal tract fibers, with those supplying the upper limb being located more centrally. Consequently, the functionality of the upper limbs can be significantly affected by central spinal cord contusion or hematoma (28). Levi *et al*. (29) proposed that intact corticospinal tracts play a critical role in hand function in primates. Additionally, neurophysiological experiments conducted by Curt et al. (30) revealed that axons controlling hand movement are more susceptible to damage compared to those controlling lower limb movement in patients with central cord syndrome (CCS). In our study, the LF group demonstrated superior performance compared to the LP group in terms of final IHMS scores. We hypothesize that this discrepancy may be attributed to the more substantial and durable increase in the space available for the spinal cord following laminectomy and fusion, which may create a more favorable environment for neural recovery. Moreover, studies have indicated that a gradual remodeling process occurs in response to laminectomy. The natural ability of the body to adapt increases glial cell proliferation and extracellular matrix deposition in the spinal cord. These changes form a more supportive environment for neural tissue growth and repair. *Pemla Jagtiani et al*. (31) reported that their umbrella review revealed that posterior cervical laminectomy with fusion had better outcomes for overall complications rate and postoperative JOA than cervical laminoplasty, but they were classified as being of weak significance. *Yoon ST et al*. (32) reported that for patients with CSM, there is low-quality evidence that suggests that laminoplasty and laminectomy and fusion procedures are similarly effective in treating CSM. For patients with ossification of the posterior longitudinal ligament, the evidence regarding the effectiveness of these procedures is insufficient. *Lee* et al. (33) reported that there is no evidence to support EL over LF in the treatment of multilevel CSM. Any superiority between EL and LF remains in question, although the LF group shows favorable long-term results. *Benek B* et al. (34) reported Laminoplasty and laminectomy with fusion improved neurological functions in patients diagnosed with CSM. Laminectomy with fusion should be the preferred choice when treating patients with preoperative axial pain as, despite expanding the spinal canal successfully, laminoplasty can also worsen the pain. In the absence of kyphotic deformity, laminoplasty should be the preferred method for treatment.

The K-line, initially proposed by Fujiyoshi and colleagues (35), is a prognostic tool used for evaluating the clinical effectiveness of posterior cervical decompression in patients with OPLL. It is defined as a linear association between the midpoints of the cervical canal from C2 to C7 on a standard lateral cervical x-ray image. Patients are classified as K-line positive when their OPLL does not extend beyond this line, while they are classified as K-line negative when their OPLL extends beyond this line. To unravel the correlation between cervical physiological curvature and clinical results, patients diagnosed with multi-segment OPLL were classified into subgroups based on the pivotal K-line index, which is linked to cervical physiological curvature (36). Our findings demonstrated that in cases where the K-line was positive, the LF group exhibited a significantly superior recovery rate (RR) compared to the LP group. Conversely, when the K-line was negative, we observed significantly higher final JOA scores, IHMS scores, and RR in the LF group compared to the LP group. The observed improvements in the outcomes of the LF group may be attributed to rigid fixation, which maintains a larger postoperative C2–C7 Cobb angle and increases the buffer space for posterior movement of the cervical cord. The maintenance of a larger postoperative C2–C7 Cobb angle allows for improved spinal alignment, reducing stress on the surrounding structures and potentially preventing further degeneration or complications.

In terms of postoperative complications, no significant differences were observed between the two groups. However, both groups exhibited a relatively high risk of C5 nerve root palsy and axial pain. C5 nerve root palsy is more frequently observed in males and typically manifests as the weakness of deltoid and biceps brachii muscles, accompanied by reduced or absent sensation in the shoulder and lateral arm. Additionally, the biceps tendon reflex may be diminished or absent. These symptoms can manifest within a few days or weeks after surgery, but they are generally transient and improve with conservative treatment. The main pathological mechanism responsible for C5 nerve root palsy is thought to be the entrapment effect exerted on the nerve root (37). With an average follow-up period of 4.1 months, Imagama et al. (38) reported that approximately 67% of patients who undergo laminoplasty and experience postoperative C5 nerve root palsy recover without surgical intervention. Postoperative C5 nerve root palsy is also a common and challenging complication after posterior laminectomy, with an overall incidence rate of 11.3%. Preexisting risk factors include intervertebral foraminal stenosis, ossification of the posterior longitudinal ligament, herniation of multi-level cervical disc, and male gender (39). Generally, C5 nerve root palsy tends to resolve within several months without specific management; however, it necessitates rehabilitation that emphasizes enhancing muscular strength and exercise to improve ROM (40).

Currently, there are few studies regarding the precise factors underlying axial pain. It is believed that variables such as cervical curvature, the extent of paravertebral muscle dissection during surgery, preoperative spinal cord compression rate, and subsequent postoperative displacement are associated with this type of pain. Moreover, a shift in the position of the spinal cord after surgery is considered a potential risk factor for axial pain. The pain can be attributed to dura mater bulging or excessive deformity affecting the cervical spinal cord. Alternatively, it can be caused by injury or necrosis affecting blood vessels responsible for the innervation of autonomic nerves. Hosono et al. (41) revealed a significantly higher prevalence of axial pain in patients who underwent laminoplasty compared to those who underwent anterior fusion (60% vs. 19%, respectively). In total, 26% of patients who underwent laminoplasty experienced postoperative neck pain with an average duration of 5.5 months; however, this condition improved within 1–1.5 years after the surgery. Levy HW et al. (42) demonstrated that patients who underwent cervical laminectomy experienced significant relief of axial pain compared to those who underwent laminoplasty, particularly when decompression was performed for more than three vertebral levels. Preserving the semispinalis cervicis muscle may prevent axial neck pain. The higher occurrence rate of axial symptoms in our study could potentially be attributed to the extensive dissection of paravertebral muscles.

In the multivariate logistic regression analysis, preoperative JOA and preoperative IHMS were identified as independent protective factors for favorable postoperative recovery (Tables 8, 9). This finding is consistent with previous studies demonstrating that better preoperative neurological status is a strong predictor of postoperative outcomes in patients with cervical myelopathy (24, 43). Notably, after adjusting for these and other potential confounders (age, preoperative ROM, and p, reoperative C2–7 Cobb angle), the surgical method (LF vs. LP) was no longer a statistically significant independent predictor of favorable recovery (*P* = 0.146 for JOA; *P* = 0.225 for IHMS).

This result necessitates careful interpretation. The observation that LF demonstrated superior outcomes in univariate analyses but not in multivariate analysis implies that the observed advantages of LF might be at least partly due to disparities in baseline characteristics between the two groups. Specifically, patients chosen for LF in this cohort had a higher prevalence of preoperative kyphosis, instability, or significant OPLL—factors that are also recognized to impact neurological recovery. Although our multivariate model accounted for several key covariates, residual confounding from unmeasured variables (e.g., socioeconomic status, rehabilitation adherence) cannot be ruled out.

The lack of a statistically significant independent effect of the surgical method does not undermine the clinical value of laminoplasty (LF). Instead, it highlights the intricacy of surgical decision-making for patients with multi-segment cervical central spinal stenosis with ossification of the posterior longitudinal ligament (CCSWOFD). The choice between LF and laminectomy with fusion (LP) should be personalized, considering patient-specific factors like cervical alignment, instability, the morphology of OPLL, and the overall surgical risk. Our findings indicate that both techniques can yield satisfactory results, yet the selection of the appropriate procedure should be based on patient characteristics rather than a universal approach.

The present study had several limitations. First, it is important to note that this retrospective study was conducted at only one institution. Second, although the standardized decision-making criteria were clearly outlined in the methods section, the choice of surgical approach was essentially determined by patient characteristics. This gives rise to selection bias and confounding by indication. Factors such as preoperative kyphosis and instability may independently affect the outcomes and limit the comparability of the groups. Third, the sample size of this study was relatively small and there was no randomization. Fourth, the follow-up duration of this study was relatively short. Moreover, multiple statistical comparisons were performed throughout this study, including *t*-tests for continuous outcomes, chi-square tests for categorical variables, and subgroup analyses based on K-line status. No adjustment for multiple comparisons was made as the analyses were considered exploratory. This approach may increase the risk of type I error (false-positive findings). Therefore, the results of secondary and subgroup analyses should be interpreted with caution. Future prospective studies with pre-specified hypotheses and appropriate correction for multiple comparisons (e.g., Bonferroni correction) are necessary to validate our findings. The heterogeneity among the patient population, which includes various pathologies like multilevel disc herniation and OPLL, may also complicate the overall outcomes. Although a subgroup analysis based on the K-line was conducted to evaluate the specific effects of OPLL, the influence of other pathologies on more comprehensive comparisons cannot be entirely excluded. These limitations could impact the generalizability of the findings. Therefore, future multicenter randomized controlled trials with larger sample sizes and longer follow-up periods are required.

## 5 Conclusion

In conclusion, both laminectomy fusion fixation and single open-door laminoplasty have demonstrated efficacy as surgical options, restoring neurological function, alleviating pain, enhancing motor ability, and reducing spinal cord edema. However, in this retrospective cohort, laminectomy fusion fixation was associated with superior outcomes compared to single open-door laminoplasty in terms of final C2–7 SVA, C2–C7 Cobb angle, JOA score, IHMS score, RR, and BASIC score. Despite the shorter surgical time and preserved ROM associated with laminoplasty, LF showed more favorable radiological and neurological outcomes in this study.

## Data Availability

The original contributions presented in the study are included in the article/Supplementary Material, further inquiries can be directed to the corresponding author.
