# Supplementary material for: Laminectomy vs. laminoplasty for treating multi-segment cervical canal stenosis combined with central cord syndrome in the absence of fracture or dislocation: a retrospective study
Source: Front Surg. 2026 Jun 8;13:1722910. doi: 10.3389/fsurg.2026.1722910 (PMC13283975; doi:10.3389/fsurg.2026.1722910)
Supplement: Supplementary file 1 [file Datasheet1.docx]

#### **1. Parameters Used for Power Analysis**

The following parameters were specified based on the observed data from our study:

| **Parameter** | **Value** | **Justification** |
| --- | --- | --- |
| Primary outcome | Final JOA score | It was chosen as the primary outcome measure because it is the most comprehensive neurological assessment tool for cervical myelopathy. |
| Effect size | Cohen's d = 0.5 (moderate effect) | Based on the observed mean difference between groups (2.30 points) and the pooled standard deviation (1.19), the calculated effect size was approximately 1.93, which surpasses the conventional threshold for a large effect (d > 0.8). |
| Alpha (α) | 0.05 | Standard significance level for hypothesis testing |
| Power (1-β) | 0.80 | Conventional threshold for adequate statistical power |
| Sample size | n = 112 (LF: 59, LP: 53) | Actual sample size of the study |
| Test family | t-test | Used for comparing two independent group means |
| Tail(s) | Two-tailed | Appropriate for hypothesis testing without directional assumption |

#### **2. Calculation and Results**

Using the observed mean difference and variability:

Observed mean difference (Δ): 2.30 points (LF: 12.98, LP: 10.68)

Pooled standard deviation (SD_pooled): √[(59-1)×1.25² + (53-1)×1.13²] / (59+53-2) = 1.19

Effect size (Cohen's d): 2.30 / 1.19 = 1.93

With these parameters, the achieved power was calculated as:

Power = 92% (1-β = 0.92, β = 0.08)

This indicates that the study had a 92% probability of detecting the observed difference in JOA scores between groups at the α = 0.05 significance level.

#### **3. Interpretation**

The post-hoc power analysis yielded the following key findings:

1. Adequate power: With a power of 92%, the study had sufficient power to detect the observed difference in the primary outcome (final JOA score). This surpasses the conventional threshold of 80%, indicating that the sample size was adequate to support the main findings. 2. Conservative interpretation: Even if we conservatively assume a smaller effect size (e.g., Cohen's d = 0.8, representing a large effect), the study would still attain approximately 85% power with the current sample size (n = 112). 3. Limitations: Post - hoc power analysis is inherently based on observed effect sizes, which might overestimate power when compared to a priori calculations. Nevertheless, the result (92% power) offers reassurance that the study was not under - powered to detect the difference in the primary outcome.
